# Glutamine metabolism and ammonia death: targeted modulation for enhanced cancer immunotherapy

**DOI:** 10.3389/fimmu.2025.1643017

**Published:** 2025-09-17

**Authors:** Fei Du, Linlin Xiao, Wang Guojun, Qian Dai, Junxin Li, Xin Zhao, Qimin Zhang, Lan Yang, Yujie Liu, Yidan Hu, Bo Wen, Jingqiu Zhou, Jie Dai, Wenhao Zhang, Zhuo Zhang

**Affiliations:** ^1^ Department of Pharmacy, The Fourth Affiliated Hospital of Southwest Medical University, Meishan, Sichuan, China; ^2^ Department of Pharmacology, School of Pharmacy, Southwest Medical University, Luzhou, Sichuan, China; ^3^ Department of Pharmacy, Zigong Fourth People’s Hospital, Zigong, China; ^4^ Department of Medical Administration, The Fourth Affiliated Hospital of Southwest Medical University, Meishan, Sichuan, China; ^5^ Key Laboratory of Luzhou City for Aging Medicine, School of Pharmacy, Southwest Medical University, Luzhou, China

**Keywords:** ammonia death, glutamine, CD8+ T cell, tumor microenvironment, immunotherapy

## Abstract

Immunotherapy has rapidly emerged as a transformative advancement in cancer treatment, becoming essential for managing diverse malignancies. Despite the remarkable clinical efficacy of immunotherapies, including immune checkpoint inhibitors (ICIs) and chimeric antigen receptor (CAR)-T cells, across various tumor types, patient responses remain heterogeneous, with some tumors developing resistance through immune evasion strategies. Presently, the investigation of cell death mechanisms is gaining momentum as a promising avenue for immunotherapy optimization. Recent studies underscore that integrating cell death pathways with immunotherapy can significantly amplify anti-tumor immune responses. Ammonia, a metabolic byproduct within the tumor microenvironment (TME), has garnered increasing interest. Specifically, emerging research suggests that ammonia, accumulating in effector T cells as a result of glutamine metabolism, induces cell death. This distinct form of cell death, termed “ammonia death,” diverges from previously characterized mechanisms. This review examines the metabolic role of glutamine in various TME cells, explores the potential regulatory links between glutamine metabolism and ammonia-induced cell death, and evaluates the feasibility of targeting ammonia-induced cell death to enhance anti-tumor immunity and improve immunotherapy outcomes.

## 1 Introduction

Immunotherapy, an established strategy that leverages the immune system to combat tumors, has seen substantial advancements in recent years (1). Among the various therapeutic approaches, ICIs and CAR-T cell therapy have emerged as the most clinically impactful, significantly improving the prognosis for various cancers (2, 3). However, challenges such as inconsistent clinical efficacy and resistance continue to impede further optimization of immunotherapy (4, 5). Consequently, the exploration of novel immunotherapeutic strategies, particularly those integrating immunotherapy with other modalities, has become a central focus in cancer immunotherapy research. Metabolic reprogramming is a hallmark of cancer, with glutamine serving as a key nutrient for both tumor proliferation and immune cell function (6). This intersection positions ammonia metabolism as a critical therapeutic node.

Glutamine, a key amino acid abundant in blood and muscle, constitutes approximately 20% of circulating amino acids (6). While traditionally considered a non-essential amino acid due to its ability to be synthesized by most mammalian tissues, glutamine becomes conditionally essential under pathological conditions, where increased demand necessitates exogenous intake (7, 8). It plays a critical role as a metabolic nutrient in the TME, fueling energy production and serving as a precursor for biosynthetic processes essential for rapid cell proliferation (9, 10). Recent evidence suggests that reprogramming glutamine metabolism is not only vital for immune cell activation and proliferation but also critical for tumor cell invasion and TME remodeling (11, 12). Despite efforts to target amino acid metabolism in cancer therapy, clinical success has thus far been limited (13). Emerging studies, however, suggest that metabolic byproducts of glutamine also play pivotal roles within the TME (14, 15).

Ammonia, a byproduct of amino acid metabolism in the TME, has gained increasing attention in recent years. Notably, recent research indicates that during immune responses, ammonia produced from glutamine metabolism in activated effector T cells serves as a nitrogen source for energy production and biosynthesis. However, excessive ammonia accumulation induces a novel form of cell death in effector T cells, termed Ammonia death (15, 16).

Distinct from traditional cell death mechanisms such as apoptosis, necrosis, and pyroptosis, ammonia-induced cell death (termed “Ammonia death”) is a novel form of cell death triggered by overload of glutamine-derived ammonia, leading to the demise of effector T cells. It is characterized by mitochondrial swelling, impaired autophagic flux, and lysosomal alkalinization (15). Studies have demonstrated that inhibiting Ammonia death significantly extends the lifespan of effector T cells, thereby enhancing the effectiveness of tumor immunotherapy (15, 16). Targeting Ammonia death presents a promising novel strategy for cancer immunotherapy.

This review consolidates the metabolic features of glutamine in tumor and immune cells within the TME, explores the regulatory relationship between glutamine metabolism and Ammonia death, and evaluates current strategies targeting Ammonia death to boost anti-tumor immunity and immunotherapy. The article aims to provide novel insights and theoretical support for future targeted immunotherapy strategies aimed at overcoming the immune-suppressive effects of Ammonia death.

## 2 The metabolic characteristics of glutamine in the TME

### 2.1 Tumor cells

The Warburg effect is a hallmark metabolic characteristic of tumor cells, wherein they preferentially rely on glycolysis to metabolize glucose and produce substantial amounts of lactate, even in the presence of oxygen. This metabolic shift inhibits the conversion of glucose into acetyl-CoA, thereby preventing its entry into the tricarboxylic acid (TCA) cycle (17, 18). To compensate for this altered metabolism, tumor cells accelerate intracellular glutamine metabolism, ensuring a continuous supply of metabolic intermediates (nitrogen and carbon sources) to sustain the TCA cycle. This phenomenon is referred to as “anaplerosis” (19, 20). Glutamine undergoes metabolism through various enzymatic processes, producing byproducts that integrate into the TCA cycle. It enters the cytoplasm *via* solute carrier (SLC) proteins (21) and is converted into glutamate by glutaminase (GLS) (22). Glutamate is then converted to α-ketoglutarate (α-KG) by glutamate dehydrogenase (GLUD) or transaminases, facilitating the replenishment of the TCA cycle (22–24). Furthermore, glutamine metabolism supports the production of nicotinamide adenine dinucleotide phosphate (NADPH) and glutathione (GSH), contributing to the maintenance of cellular redox homeostasis (25, 26).

Glutamine metabolism is pivotal for tumor cell survival and proliferation. Studies indicate that many tumor cells upregulate SLC family transporters to enhance glutamine uptake and stimulate the activity of enzymes involved in glutamine metabolism, thus sustaining proliferative demands (27, 28). Among these, SLC1A5 (also known as ASCT2) serves as the primary transporter for glutamine, with its overexpression strongly linked to the growth, metastasis, and drug resistance of several cancers, including breast, lung, liver, and gastric cancers (29–31). Other transporters, such as SLC7A5, SLC38A2, and SLC7A11, also play contributory roles in glutamine and glutamate transport (32–34).

**Figure 1:**
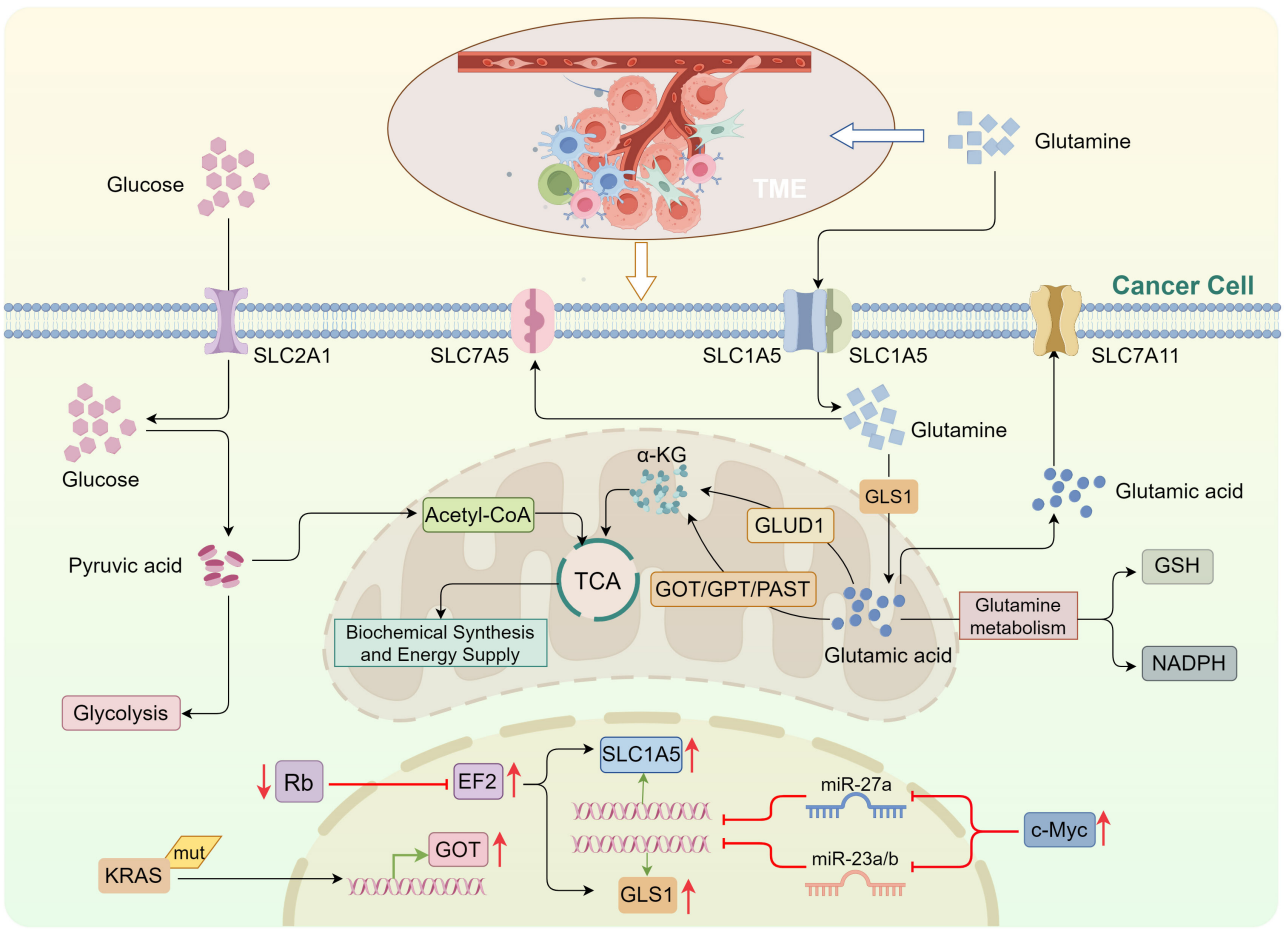
Glutamine metabolism in tumor cells replenishes the tricarboxylic acid cycle. Tumor cells primarily rely on glycolysis to metabolize glucose, impairing its conversion into acetyl-CoA for entry into the TCA cycle. To compensate, tumor cells enhance intracellular glutamine metabolism. Upon entering the cell *via* solute carrier (SLC) transporters, glutamine is converted into glutamic acid by GLS. Glutamic acid is then converted into α-KG, which enters the TCA cycle through two distinct pathways: *via* GLUD, and *via* transaminases, including GOT, GPT, and PSAT. c-Myc suppresses miR-27a and miR-23a/b expression, upregulating SLC1A5 and GLS1, respectively. E2F activation also promotes the expression of SLC1A5 and GLS1. KRAS mutations can induce GOT expression. *TCA, tricarboxylic acid; GLS, glutaminase; GLUD, glutamate dehydrogenase; GOT, glutamate oxaloacetate transaminase; GPT, glutamate pyruvate transaminase; PSAT, phosphoserine aminotransferase; α-KG, α-ketoglutarate*.

As a rate-limiting enzyme in the “anaplerosis” pathway, GLS catalyzes the breakdown of glutamine into free ammonia and glutamate (22). Studies have demonstrated that GLS1 is overexpressed in various tumor types and is closely associated with advanced tumor stages, high invasiveness, metastatic potential, and poor clinical prognosis (35, 36). Similarly, GLUD1, a key enzyme in glutamine catabolism, is highly expressed in many tumors (37). Research indicates that GLUD1 converts glutamate into α-KG, releasing substantial amounts of free ammonia in the process (38, 39). Under the influence of GLUD1 and enzymes such as glutamine synthetase (GS), the ammonia is incorporated into amino acid synthesis pathways, thereby promoting tumor cell proliferation and migration (40, 41). Alternatively, glutamate can be converted into α-KG by transaminases, such as glutamate-pyruvate transaminase 2 (GPT2), without producing ammonia. GPT2 catalyzes the reaction between glutamate and pyruvate, generating α-KG and alanine, which accelerates liver cancer cell proliferation (42). GPT2 is highly expressed in various cancers and correlates with patient prognosis (43, 44) (**Figure 1**).

The oncogene c-Myc is a major regulator of glutamine utilization in tumor cells (45). c-Myc enhances glutamine uptake by binding to the promoter regions of glutamine transporters or repressing the expression of miR-27a, which promotes SLC1A5 upregulation (46, 47). Furthermore, c-Myc upregulates GLS1 expression by transcriptionally inhibiting miR-23a/b, thereby enhancing glutamine metabolism (48). c-Myc also influences glutamine metabolism indirectly by modulating the expression of miR-15a and miR-16 (49, 50). Recent studies have highlighted that c-Myc can also regulate glutamine metabolism through the upregulation of long non-coding RNAs (lncRNAs) such as H19 and MALAT1 (51, 52). Similarly, KRAS mutations in tumor cells promote glutamine metabolism by inducing the expression of glutamate oxaloacetate transaminase (GOT), contributing to the maintenance of tumor cell proliferation and survival (53, 54). Conversely, the loss of tumor suppressor proteins from the Rb family activates E2F transcriptional activity, directly upregulating the expression of SLC1A5 and GLS1, thus facilitating glutamine uptake and utilization (55) (**Figure 1**).

### 2.2 T cells

**Figure 2:**
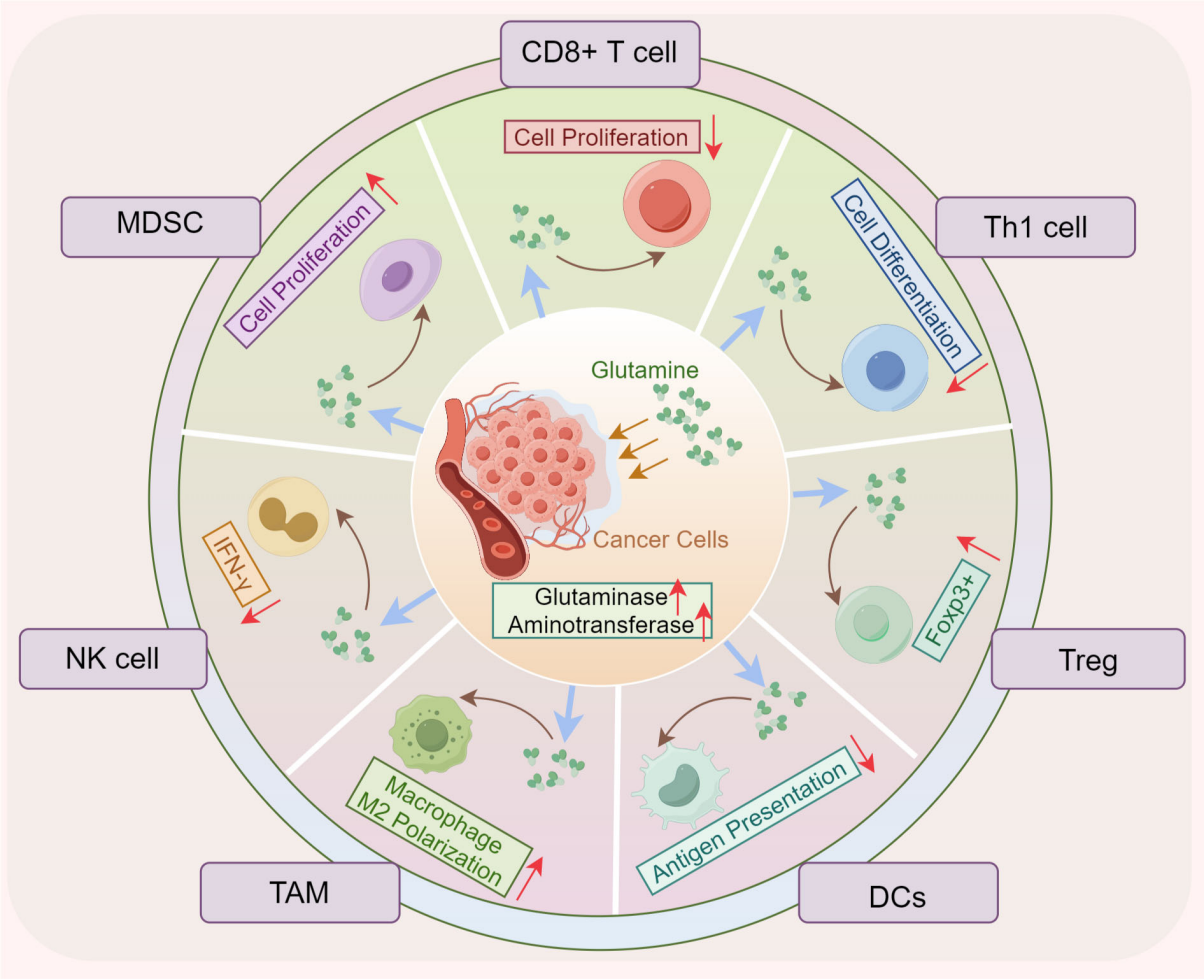
Glutamine metabolism promotes tumor immune evasion. The enhanced uptake of glutamine by tumor cells reduces its availability for infiltrating immune cells, impairing the proliferation and differentiation of CD8+ and CD4+ T cells, reducing antigen presentation by DCs, and diminishing natural killer (NK) cell antitumor activity. In contrast, the proliferation and polarization of MDSCs and TAMs are paradoxically increased. *DCs, dendritic cells; Treg, regulator T cells; NK, natural killer; MDSC, myeloid-derived suppressor cells; FOXP3, forkhead box P3; TAM, tumor-associated macrophages*.

Immune cells are essential components of the TME, playing pivotal roles in tumor initiation, progression, immune evasion, and metastasis (56, 57). Increasing evidence suggests that while immune cells are tasked with mounting anti-tumor responses, they also contribute to tumor progression through various mechanisms (57, 58). Within the TME, tumor cells competitively consume glutamine, depriving immune cells of this vital nutrient. This results in restricted glutamine metabolism in immune cells, diminishing their anti-tumor efficacy and promoting immune evasion (57) (**Figure 2**). The metabolic profiles of immune cells significantly vary based on their differentiation and functional states.

#### 2.2.1 CD8+ T cells

CD8+ T cells, key immune effector cells in the TME, are involved in tumor clearance, as well as in responding to viral infections and other pathological conditions (56). Glutamine metabolism is vital for the activation, proliferation, effector functions, and formation of immune memory in CD8+ T cells (59, 60). Intriguingly, *in vitro* studies have demonstrated that glutamine deprivation can promote the differentiation of memory CD8+ T cells, facilitating the development of long-lasting immune memory (61, 62). Conversely, *in vivo* inhibition of glutamine metabolism helps prevent CD8+ T cell exhaustion, sustaining their long-term effector functions (63). Recent research has elucidated the mechanisms underlying these effects: CD8+ T cells regulate isocitrate dehydrogenase 2 (IDH2) activity in the mitochondria, reducing glutamine through carboxylation and generating metabolites such as succinate. These metabolites not only provide energy for T cells but also “lock” them into a terminal effector differentiation state through epigenetic mechanisms, including histone modifications. Thus, inhibiting glutamine metabolism enhances memory T cell formation (62, 64). Moreover, inhibiting glutamine metabolism alleviates the long-term nutrient limitations and immune suppressive pressures within the TME, aiding in metabolic adaptation and preventing CD8+ T cell exhaustion (65). Additionally, reduced glutamine metabolism can lower the expression of immune checkpoint molecules (e.g., PD-1) on CD8+ T cells, thereby mitigating immune evasion within the TME (32).

#### 2.2.2 CD4+ T cells

Upon antigenic stimulation, CD4+ T cells undergo metabolic reprogramming, activating pathways critical for their proliferation and differentiation (66, 67). Glutamine plays a central role in this process. Studies indicate that CD4+ T cells comprise distinct subpopulations, and modulation of glutamine metabolism profoundly influences their differentiation and effector functions (68, 69). In glutamine-deprived conditions, naïve CD4+ T cells preferentially differentiate into Foxp3+ regulatory T (Treg) cells, known for their immunosuppressive activity (70). Conversely, supplementation with exogenous α-KG or its analogs enhances mTORC1 activation in naïve CD4+ T cells, upregulating the transcription factor T-bet and driving Th1 differentiation (71). Glutamine metabolism thus promotes cytokine production, including interferon-γ (IFN-γ) and IL-2, through the activation of mTORC1 and c-Myc, contributing to potent anti-tumor responses (72). Additionally, glutamine metabolism regulates CD4+ T cell differentiation *via* epigenetic mechanisms. The enzyme IDH1/2 generates 2-hydroxyglutarate (2-HG), which inhibits α-KG’s metabolic functions (73). Inhibition of glutamate conversion to α-KG prevents 2-HG production in Th17 cells, thereby affecting DNA demethylase activity and reducing FOXP3 gene locus methylation, promoting Th17 to Treg cell differentiation (74). Notably, GLS deficiency impairs naïve T cell activity and Th17 differentiation, while enhancing T-bet expression to foster Th1 and CD8+ T cell differentiation and function (75).

### 2.3 Other cells

In addition to tumor cells and T cells, the TME also contains various other cell types that play important roles in tumor initiation, progression, immune evasion, and response to therapy.

As an indispensable component of the TME, tumor-associated macrophages (TAMs) also exhibit a high dependence on glutamine metabolism (58). Research has demonstrated that glutamine not only supports the TCA cycle in TAMs but also influences their polarization (76, 77). M2-like TAMs, compared to their M1-like counterparts, exhibit elevated expression of glutamine transporters and metabolic enzymes. Inhibition of glutamine synthetase can trigger the conversion of M2-like TAMs to the more inflammatory M1-like phenotype, thus impeding tumor metastasis (77, 78). TAMs, particularly M2 macrophages, rely on glutamine metabolism to sustain cell proliferation and immune suppression (79). Recent findings indicate that chemotherapy-responsive macrophages secrete interleukin (IL)-18, which upregulates the expression of L-type amino acid transporter 2 (LAT2) in osteosarcoma cells. This enhances leucine and glutamine uptake, activating mTORC1 and promoting c-Myc-mediated transcription of CD47, which inhibits macrophage phagocytosis and facilitates immune evasion (80). Another study highlights that competitive uptake of extracellular glutamine by clear cell renal carcinoma activates hypoxia-inducible factor 1α (HIF-1α) in tumor-infiltrating macrophages, inducing IL-23 secretion (81). IL-23 subsequently activates Treg proliferation and stimulates the release of immunosuppressive cytokines, including IL-10 and transforming growth factor (TGF)-β, thus suppressing cytotoxic lymphocyte effector functions (81).

Myeloid-derived suppressor cells (MDSCs) are a diverse subset of bone marrow-derived cells, typically generated in response to stress stimuli such as tissue injury and inflammation (82, 83). MDSCs play a pivotal role in maintaining immune tolerance and preventing excessive immune responses (82). In the TME, competition for glutamine between tumor cells and myeloid cell SLC1A5 transporters significantly promotes MDSC generation and recruitment, while inhibiting the formation of anti-tumor inflammatory TAMs, thus impairing anti-tumor immunity (79). This occurs as glutamine scarcity in the TME induces endoplasmic reticulum stress in myeloid cells, activating the IRE1α/XBP1 signaling pathway, which upregulates the expression of GPR109A, driving their immunosuppressive polarization (79). Additionally, glutamine metabolism regulates MDSC suppressive function *via* the glutamate-NMDA receptor axis (84).

Dendritic cells (DCs) are essential to the immune system, particularly in antigen presentation (85). Recent studies have emphasized the central role of glutamine metabolism in DC function, particularly in type 1 conventional dendritic cells (cDC1s) (33). In the TME, tumor cells and cDC1s compete for glutamine uptake through the SLC38A2 transporter (33, 86). This glutamine deprivation in cDC1s diminishes their antigen presentation capacity *via* the FLCN-Tfeb signaling pathway, ultimately impairing anti-tumor T cell responses (33).

Natural killer (NK) cells, key effectors in the immune defense against viruses and tumors, rely on glutamine for proper function (30, 87). Research shows that SLC7A5 serves as the primary glutamine transporter in activated NK cells. Glutamine uptake activates mTORC1, increasing c-Myc expression and promoting NK cell proliferation (30). However, when tumor cells competitively deplete glutamine, activated NK cells undergo glutamine scarcity, leading to reduced glycolysis and oxidative phosphorylation, which suppresses IFN-γ production (30, 88). Although glutamine is crucial for maintaining c-Myc expression, it is not a primary metabolic fuel for NK cells. Studies suggest that while glutamine metabolism inhibitors can significantly impact tumor cells, they do not suppress NK cell metabolism or functional responses (30).

## 3 Glutamine metabolism and ammonia death

### 3.1 Ammonia accumulation dynamics: metabolic sources vs. clearance pathways

Ammonia metabolism in the TME has garnered significant attention in recent years, emerging as a critical area of study in both immunology and oncology (14). As a major metabolic byproduct, ammonia plays multifaceted roles in the TME, influencing tumor progression, immune evasion, and therapeutic responses (89, 90). Beyond serving as a nitrogen source for nucleic acid and protein synthesis, ammonia accumulates in the TME through various metabolic pathways (14). Rapid tumor cell growth and proliferation in the TME often coincide with shifts in metabolic pathways, with ammonia accumulation tightly linked to cellular metabolic reprogramming, protein degradation, and the urea cycle (14, 91). Ammonia concentrations in the TME are elevated compared to normal tissues, with distinct regional distribution patterns (14).

**Figure 3:**
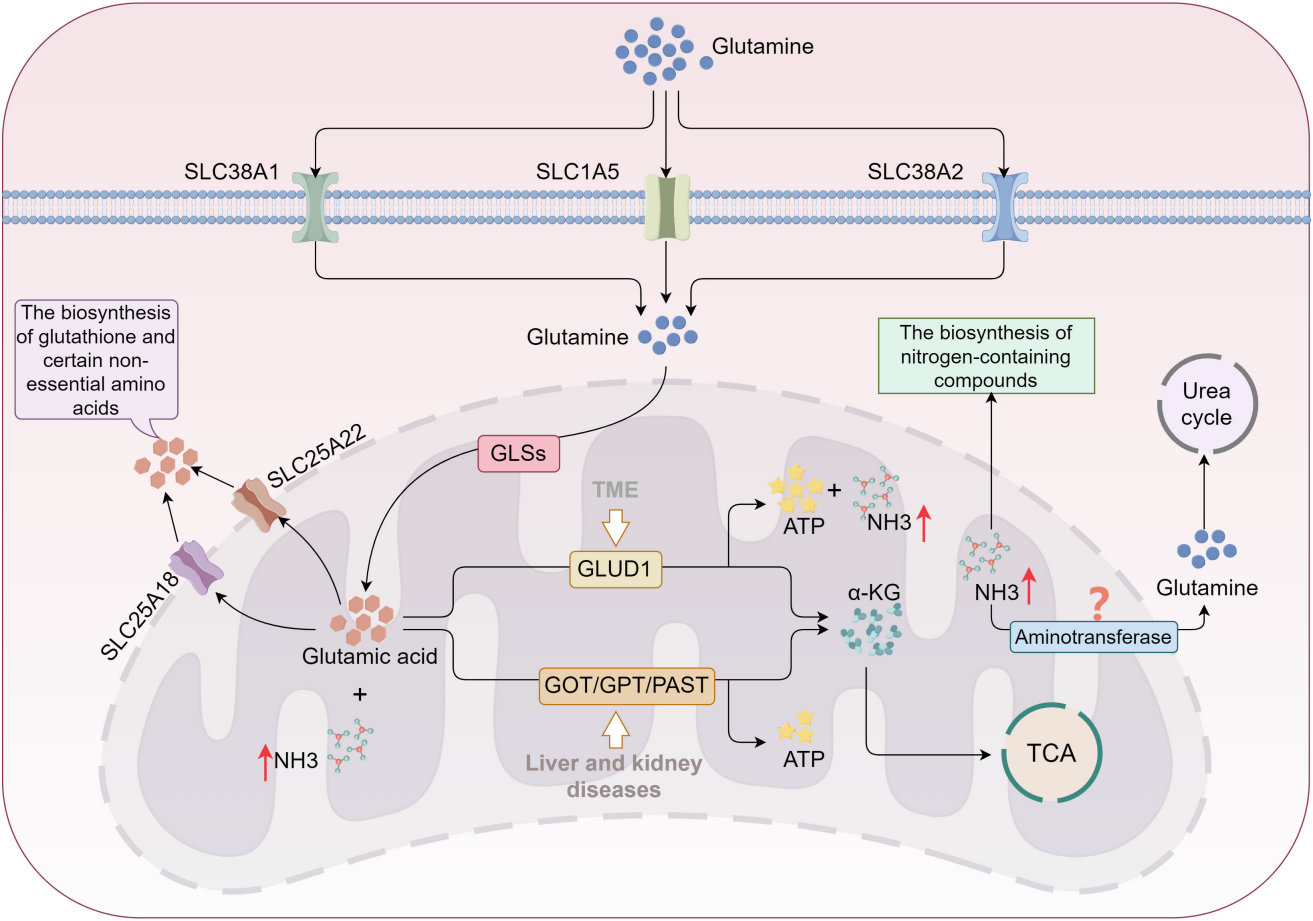
Glutamine metabolism promotes ammonia accumulation in tumor cells. Glutamine is transported into cells *via* SLC1A5 and SLC38A1/2, then converted into glutamic acid by GLS. A portion of glutamic acid is directed toward biosynthesis in the cytoplasm, while most is converted into α-KG and enters the TCA cycle through two distinct pathways. In kidney and liver diseases, glutamic acid primarily follows the transaminase pathway; however, in the TME, it favors the GLUD1 pathway, resulting in significant ammonia production. The TME downregulates enzymes involved in the urea cycle, destabilizing the tumor cells’ ability to clear ammonia and leading to its accumulation. *TCA, tricarboxylic acid; GLS, glutaminase; α-KG, α-ketoglutarate; GLUD, glutamate dehydrogenase; GOT, glutamate oxaloacetate transaminase; GPT, glutamate pyruvate transaminase; PSAT, phosphoserine aminotransferase.*.

Both tumor and immune cells exhibit increased amino acid demands to sustain their proliferation and survival (92, 93). The primary source of ammonia within cells is the deamination of amino acids (94). Glutamine, a key nitrogen and energy donor for tumor cells, is imported into the cytoplasm *via* the transporters SLC1A5, SLC38A1, and SLC38A2. It is subsequently transferred to the mitochondrial inner membrane *via* a variant of SLC1A5 (29, 95). Within the mitochondria, glutamine is converted into glutamate by glutaminases (GLS1, GLS2, GAC), releasing free ammonia (22, 96). The glutamate produced can either return to the cytoplasm *via* SLC25A18 and SLC25A22 transporters for the biosynthesis of GSH and non-essential amino acids (e.g., aspartate, alanine, arginine) or undergo complete oxidation or conversion into α-KG by mitochondrial transaminases (e.g., GPT2, GOT2) (97, 98). Pathway variations in the conversion of glutamine to α-KG occur under different pathological conditions. In liver and kidney diseases, such as hepatitis, cirrhosis, and renal failure, glutamate primarily converts to α-KG through transaminase pathways (99). In contrast, within the TME, factors like hypoxia and oxidative stress activate the GLUD pathway as a result of metabolic reprogramming. The oxidative metabolism of glutamate through GLUD activity generates substantial adenosine triphosphate (ATP), with ammonia and carbon dioxide as byproducts (93), significantly contributing to ammonia buildup in the TME (**Figure 3**). Isotope tracing studies have confirmed that the majority of ammonia in the TME is derived from the amino group of glutamines (15), further underscoring that ammonia accumulation is predominantly driven by glutamine metabolism.

Ammonia clearance is essential for maintaining cellular homeostasis and supporting growth and proliferation. Under normal conditions, the urea cycle (ornithine cycle) serves as the primary mechanism for ammonia detoxification, particularly in hepatocytes (100). In liver cells, urease-related enzymes in the mitochondria—such as carbamoyl-phosphate synthetase I, ornithine transcarbamylase, and argininosuccinate synthetase—convert ammonia into urea, which is then transported to the kidneys *via* the bloodstream and excreted in urine (100). Extracellular tissues can also incorporate ammonia into glutamine *via* transaminases, with glutamine then transported through the circulatory system to the liver to participate in the urea cycle (101). While the urea cycle is not commonly present in most tissue cells, many cells retain some capacity to clear ammonia (101). Non-hepatic cells can utilize alternative synthetic pathways for nitrogen-containing compounds, such as purines and pyrimidines, to clear ammonia, although these mechanisms are less efficient and cannot completely eliminate ammonia (102). In the TME, despite various ammonia clearance mechanisms, rapidly proliferating cells, particularly tumor and immune effector cells, have a heightened demand for α-KG derived from glutamine breakdown to fuel the TCA cycle, leading to substantial free ammonia production (14). Research indicates that tumor cells downregulate the expression of urea cycle enzymes (e.g., ornithine aminotransferase), reducing ammonia conversion into urea (103). This disruption in ammonia production and clearance balance contributes to the accumulation of ammonia. Furthermore, although lysosomes, as crucial organelles for degrading and recycling cellular metabolic waste, can transport ammonia into the lumen via the Rhesus glycoprotein C (RHCG) to reduce the concentration of free ammonia in cells, this clearance pathway is biologically limited. Prolonged and sustained ammonia stimulation may instead impair lysosomal degradative function (15, 104). Consequently, ammonia accumulation is not a transient phenomenon; rather, ammonia-induced cellular damage is more closely associated with chronic conditions, such as cancer (**Figure 3**).

### 3.2 Mechanisms of ammonia death

Ammonia is generally regarded as a cytotoxin, and elevated blood levels lead to hyperammonemia, impairing cellular function (105, 106). In the TME, excessive ammonia accumulation triggers endoplasmic reticulum (ER) stress, activating ER stress sensors such as IRE1 and PERK. This activation promotes reactive oxygen species (ROS) production and induces oxidative stress, resulting in significant cellular and tissue toxicity (107).

Ammonia accumulation is closely associated with protein synthesis and degradation processes (90, 106, 108). Excess ammonia disrupts cellular protein metabolism. The mTOR pathway, a key regulator of cell growth, proliferation, and metabolism (90), is modulated by ammonia levels. Under normal conditions, ammonia activates amino acid sensors, enhancing mTORC1 activity and promoting protein synthesis. However, at high concentrations, ammonia and increased oxidative stress initiate negative feedback on mTORC1. Through modulation of upstream factors like AMPK, ammonia inhibits mTOR activity, thereby reducing protein synthesis (106). Regarding protein degradation, ammonia primarily influences this process *via* the ubiquitin-proteasome system (UPS) and autophagy (108). Excess ammonia enhances the activity of ubiquitin ligases, such as E3 ubiquitin ligase, facilitating protein degradation. It can also activate cellular stress responses, including plasma membrane and ER stress (e.g., UPR), accelerating the removal of damaged proteins (108).

Ammonia accumulation has a dual effect on autophagy (15, 109, 110). At low to moderate levels, ammonia activates the AMPK signaling pathway, enhancing autophagy to clear ROS-induced damage and dysfunctional proteins, helping cells adapt and survive (110). However, at high levels of ammonia, it may excessively activate the mTORC1 pathway or interfere with the autophagy initiation complex, inhibiting autophagic function. In this scenario, the failure to effectively clear damaged proteins and organelles leads to cellular dysfunction and eventual cell death (110).

**Figure 4:**
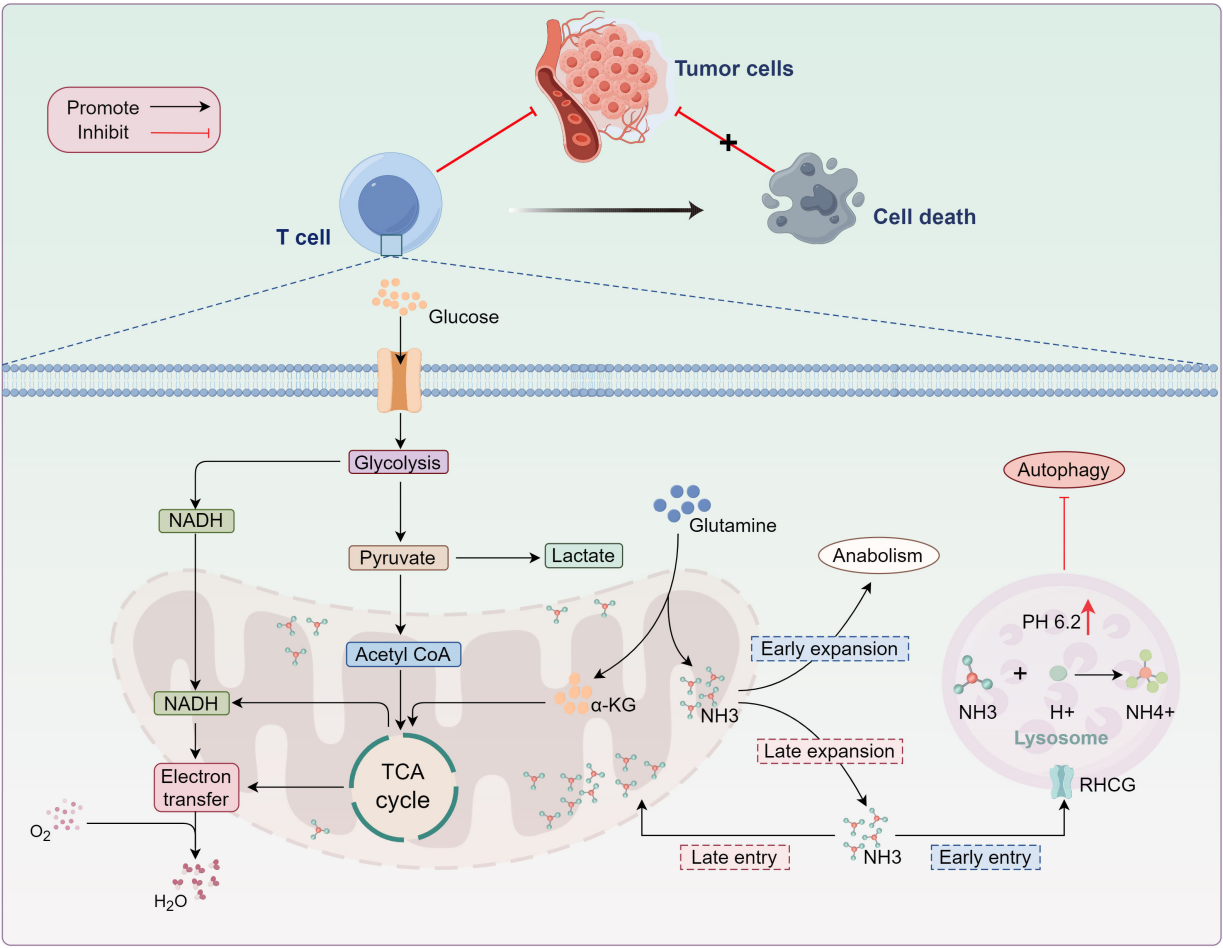
Ammonia-induced T-cell death. As glutamine metabolism progresses, ammonia (NH3) produced during glutamine decomposition exits the mitochondria and enters the lysosome, where it reacts with H+ to form NH4+. This reaction results in lysosomal alkalinization, terminating ammonia storage in the lysosome. Excess ammonia accumulation in the cytoplasm disrupts mitochondrial function. While damaged mitochondria are typically cleared *via* autophagy, lysosomal alkalinization inhibits enzymatic activity, obstructing the removal of dysfunctional mitochondria and ultimately inducing effector T-cell death. *TCA, tricarboxylic acid; α-KG, α-ketoglutarate; NADPH, nicotinamide adenine dinucleotide phosphate*; *RHCG*, *rhesus glycoprotein C.*.

In 2024, Professor Huang’s team uncovered a novel form of T cell death, termed “Ammonia death” (15, 16). This study, for the first time, explored ammonia-induced cell death from a metabolic perspective, offering a new explanation for the rapid exhaustion of effector T cells (Teffs) following anti-tumor activity. Ammonia death is a distinct form of cell death driven by lysosomal and mitochondrial damage due to ammonia accumulation, characterized by lysosomal alkalization and mitochondrial swelling (15, 16). In contrast to traditional cell death mechanisms such as apoptosis and T cell exhaustion, the accumulation of ammonia from glutamine metabolism is identified as the primary trigger for “Ammonia death” (15, 16). During tumor immune responses, rapidly proliferating T cells become highly dependent on glutamine metabolism, leading to the release of large quantities of ammonia in the mitochondria (15, 16). However, when ammonia accumulates excessively in lysosomes, it raises the lysosomal pH, which not only damages the lysosomal membrane but also halts the storage of ammonia in the lysosome, resulting in its release back into the cytoplasm (15). As lysosomes lose their capacity to store ammonia, it accumulates around the mitochondria, triggering mitochondrial dysfunction and initiating oxidative stress (15, 104). This dual damage to lysosomes and mitochondria due to excessive ammonia accumulation ultimately promotes cell death (15). Furthermore, autophagy is one of the crucial mechanisms for maintaining intracellular homeostasis, preserving normal cellular function by clearing damaged or unnecessary cellular components (15). However, due to lysosomal impairment, excess ammonia accumulates in mitochondria, leading to increased mitochondrial mass. Although autophagosomes can form in the cytoplasm, autophagic flux appears to be interrupted. The blockade of autophagy prevents the efficient clearance of damaged mitochondria, which further exacerbates cellular dysfunction and ultimately accelerates cell death (15, 16) (**Figure 4**).

### 3.3 Potential relationship between ammonia death and others

While Ammonia death differs in morphology and mechanism from other cell death forms, excessive ammonia promotes the generation of ROS, inducing oxidative stress (15, 16). This suggests that ammonia-induced Ammonia death is not an isolated mode of cell death but may be interconnected with other well-established cell death processes, such as apoptosis, autophagic cell death, and necrosis.

Apoptosis, a form of programmed cell death, is typically executed through an intrinsic self-destruction pathway that involves caspase family activation (mainly caspases 3 and 7), alterations in the cell membrane, and DNA fragmentation (111). Mitochondrial dysfunction caused by ammonia accumulation creates an oxidative stress environment, a critical condition for activating the caspase pathway (112). At low ammonia concentrations, cells typically undergo a sub-lethal apoptotic response, a mechanism designed to eliminate mildly damaged cells (113). However, at higher concentrations, ammonia induces significant mitochondrial damage, compromising mitochondrial membrane integrity and promoting the release of cytochrome c and other pro-apoptotic molecules (such as SMAC/DIABLO) into the cytoplasm. This activates the apoptosome, leading to caspase-9 and caspase-3 activation, which triggers the apoptotic program (112).

Autophagy is a process in which cells degrade and recycle damaged organelles and macromolecules, typically activated in response to nutrient deprivation or stress (114). The relationship between Ammonia death and autophagy is complex. As mentioned earlier, varying ammonia concentrations yield different effects on autophagy (109, 115). In the early stages of autophagy, ammonia triggers oxidative stress and mitochondrial damage, stimulating the autophagic machinery to clear damaged mitochondria and oxidative damage. However, as ammonia accumulates to higher concentrations, it may disrupt the initiation and degradation stages of autophagy (e.g., inhibiting autophagosome-lysosome fusion), impairing autophagic flux and promoting autophagic cell death (109, 115).

Cell necrosis is an acute, non-programmed form of cell death typically triggered by membrane rupture, leading to the release of cellular contents and subsequent inflammation (116). The link between ammonia-induced cell death and necrosis is likely due to the local accumulation of ammonia at high concentrations, which disrupts cell membrane integrity and initiates necrotic responses such as cell swelling, membrane rupture, and the leakage of intracellular components into the extracellular space (117, 118). This leakage contributes to local inflammation and tissue damage.

Pyroptosis, a programmed cell death process driven by iron ions and lipid peroxidation, has been implicated in various pathological conditions (119). While direct evidence connecting ammonia-induced cell death with pyroptosis is currently lacking, several studies suggest that ammonia accumulation exacerbates oxidative stress, potentially activating inflammasomes and thereby promoting pyroptotic cell death (119, 120).

In conclusion, ammonia-induced cell death represents a distinct mode of cellular demise caused by ammonia accumulation, overlapping with and interacting with apoptosis, autophagy, necrosis, and other forms of cell death. Its unique characteristic is the metabolic reprogramming of the cell and the specialized response to ammonia buildup, positioning ammonia death as a promising target for future immunotherapeutic strategies.

## 4 Ammonia death in TME

### 4.1 Immune cells with ammonia death

Upon antigen stimulation, CD8+ naïve T cells differentiate into effector cells, with over 95% undergoing rapid cell death following antigen clearance in a process known as T cell contraction (121). This death of effector T cells is essential for maintaining immune homeostasis and preventing autoimmune responses. However, the precise mechanisms underlying effector T cell death have remained unclear. Professor Huang Bo’s team demonstrated that the ketone-body-gluconeogenesis-glycogen metabolic pathway activates the pentose phosphate pathway to reduce intracellular ROS, promoting the formation and maintenance of CD8+ T cell memory (122, 123). Furthermore, they showed that the urea cycle plays an active role in reducing intracellular ammonia levels, which supports the long-term survival of memory T cells (104).

Recent studies have highlighted ammonia’s critical role in effector CD8+ T cell death (15). While glutamine metabolism provides essential energy for CD8+ T cells during immune responses, contributing carbon and nitrogen for biosynthesis, its metabolic byproduct, ammonia, gradually accumulates within the cells, ultimately leading to their demise (15, 16). To prevent ammonia-induced cell death and extend the survival of CD8+ T cells, at least three strategies have been proposed. First, since glutamine metabolism is the precursor to ammonia accumulation, inhibitors such as 6-diazo-5-oxo-L-norleucine (DON) or CB-839, a non-competitive allosteric inhibitor of GLS1, can significantly reduce intracellular ammonia levels (35, 124). Both DON and CB839 have been shown to promote Teff survival, upregulate CD25, IFN-γ, and TNF-α, and enhance the formation of memory precursor effector cells (KLRG1-CD127+) (125–127). Second, removing excess ammonia from the cell provides an alternative approach to mitigate ammonia-induced cell death. Ammonia scavengers, such as phenylbutyrate (a prodrug of phenylacetic acid), reduce ammonia levels by promoting its conversion into phenylacetyl glutamine, which is excreted in urine, thus mitigating its toxic effects (128). Additionally, upregulating the key urea cycle enzyme, carbamoyl-phosphate synthetase 1 (CPS1), which is expressed at low levels in CD8+ T cells, can significantly lower intracellular ammonia concentrations (15, 129). Third, because ammonia-induced damage to lysosomes and mitochondria directly triggers cell death, accelerating the clearance of damaged organelles or enhancing autophagy offers a promising strategy to rescue T cells. Restoring lysosomal function, for example, by activating the transcription factor TFEB using compounds like Torin1 or Metformin to upregulate lysosomal gene expression, promotes lysosome biogenesis (130–132). When lysosomal function is severely impaired, lysosome-independent degradation pathways, such as those activated by Bortezomib, a proteasome modulator, can enhance ubiquitination and proteasomal activity, compensating for defective lysosomal function and facilitating the removal of damaged proteins and organelles, thus improving immunotherapy efficacy (133) (**Table 1**). In summary, targeting ammonia-induced cell death can significantly prolong the survival of CD8+ T cells in the TME, enhancing their anti-tumor activity. This approach offers a promising direction for future cancer immunotherapy.

**Table 1:** Potential strategies for inhibiting ammonia death.

Category	Drugs	Mechanism	Reference
GLS1 Inhibitors	BPTES	Selectively inhibits GLS1	(157)
	CB-839	Selectively inhibits GLS1	(35, 205)
	IACS-6274	Selectively inhibits GLS1	(160)
	IPN60090	Selectively inhibits GLS1	(161)
	DON	Competes for glutamine-utilizing enzymes	(206, 207)
	JHU-083	Competes for glutamine-utilizing enzymes	(208)
	sirpiglenastat	Competes for glutamine-utilizing enzymes	(209, 210)
Ammonia Remover	4PBA	Enhances the expression of urea cycle-related enzymes (e.g., CPS1)	(211, 212)
	C381	Reduces lysosomal pH	(213, 214)
	SYNB1020	Converts intestinal ammonia into arginine	(215)
Enhancing autophagy	LH2-051	Inhibits the interaction between dopamine transporter (DAT) and CDK9, reducing TFEB phosphorylation	(216, 217)
	Rapamycin	Blocks the mTORC1 pathway	(218, 219)
	TPCA-1	Dual inhibition of STAT3 and NF-κB pathways	(220, 221)
	EBSS	Activates the AMPK pathway and inhibits the mTOR pathway	(222, 223)
	Lithium Chloride	Inhibits IMPase or modulates lysosomal acidification	(224, 225)
	Tubastatin A	Selectively inhibits HDAC6	(226, 227)
Others	V-9302	Competitively inhibits glutamine uptake	(60, 228)
	Arginine	Stimulates urea production	(104, 229)
	Sodium/potassium glutamate	Combines with ammonia to generate glutamine	(230, 231)

BPTES, bis-2-(5-phenylacetamido-1,2,4-thiadiazol-2-yl) ethyl sulfide; DON, 6-diazo-5-oxo-L-norleucine; 4PBA, 4-Phenylbutyric acid; EBSS, earle’s balanced salt solution; IMPase, inositol monophosphatase.

Although current experimental research on ammonia-induced cell death primarily focuses on CD8+ T cells, the immune system operates as a complex network. As discussed in the previous section, glutamine metabolism, which leads to ammonia accumulation, is active in a variety of immune cells. This metabolic pathway facilitates ammonia buildup in these cells (68, 69, 76, 77, 79). Furthermore, research indicates that CPS1, the key enzyme in the urea cycle, is predominantly expressed in hepatocytes and certain renal cells, with low or absent expression in most immune cells due to the urea cycle’s inactivity in these cells (134, 135). This limitation further contributes to ammonia accumulation in other immune cells. Notably, effector CD4+ T cells, including Th1, Th2, and Th17 cells, play vital roles in short-term immune responses. Following activation and execution of their effector functions, the majority of effector CD4+ T cells undergo programmed cell death (apoptosis), making them unsuitable for long-term immunity (136, 137). Therefore, it is reasonable to speculate that ammonia-induced cell death may not be a form of cell death unique to CD8^+^ T cells. Although CD8^+^ T cells may be more susceptible to ammonia under certain tumor microenvironments or immunosuppressive conditions, other immune cells (such as CD4^+^ T cells and macrophages)may also undergo cell death due to ammonia overload.

### 4.2 Tumor cells with ammonia death

Research on ammonia-induced cell death has primarily focused on immune cells, particularly CD8+ T cells (15, 16). Inhibiting ammonia-induced cell death appears to significantly extend the lifespan of CD8+ T cells, thereby enhancing long-term immune efficacy (15). However, whether tumor cells undergo ammonia-induced cell death has yet to be investigated. Current evidence suggests that the primary mechanism triggering ammonia-induced cell death involves the excessive accumulation of ammonia, a byproduct of glutamine metabolism, which leads to lysosomal and mitochondrial damage (15, 16). As a metabolic byproduct, ammonia plays a critical role not only in immune cells but also in tumor cells (138, 139).

In the TME, the rapid proliferation of tumor cells is often associated with increased metabolic activity and competition for nutrients, including glutamine, from the surrounding environment. Consequently, tumor cells typically exhibit higher glutamine metabolic activity than immune cells (140). Studies indicate that, under normal conditions, ammonia produced by glutamine metabolism can promote tumor progression. For instance, ammonia serves as a key activator of lipogenesis, which is a prominent marker of cancer progression and metastasis (141). Lipogenesis is regulated by Sterol Regulatory Element-Binding Proteins (SREBPs) (141, 142). Cheng et al. demonstrated that glutamine is essential for SREBP activation and lipogenesis. While glucose is required for the N-glycosylation-mediated stabilization of SCAP, glutamine is necessary for the nuclear translocation of SREBP (90). Further studies revealed that ammonia, rather than glutamate or α-KG, is the primary activator of SREBP cleavage (90). Interestingly, gene inhibition and CB-839, an inhibitor of GLS activity, can eliminate SREBP activation, suggesting that ammonia derived from GLS is a critical mediator of lipogenesis (90, 142). Additionally, studies have shown that in certain tumors, blocking glutamine uptake leads to cell death, a phenomenon known as “glutamine addiction” (143, 144). Consequently, recent studies have increasingly focused on inhibiting glutamine metabolism in tumor cells as a strategy to suppress tumor progression.

Given the established mechanisms of ammonia-induced cell death in CD8+ T cells, inducing ammonia-induced cell death in tumor cells through excessive ammonia accumulation presents a promising avenue for cancer immunotherapy research. CPS1 catalyzes the reaction between ammonia and carbon dioxide to form carbamoyl phosphate, the first step in urea synthesis (134, 145). This process helps maintain metabolic balance by clearing ammonia from cells and mitigating oxidative damage (134, 145). Research indicates that CPS1 is predominantly expressed in liver, kidney, and a small number of intestinal epithelial cells (134, 135, 146). In the TME, CPS1 expression varies across cancer types and is highly expressed in liver, pancreatic, kidney, colorectal, breast cancers, and non-small cell lung cancer (135, 146–148). Several small-molecule CPS1 inhibitors have been identified, showing potential in inhibiting CPS1 activity and inducing tumor cell death under experimental conditions (135, 146, 149–151). However, these inhibitors remain in the early stages of research and have yet to be extensively validated in clinical cancer treatments. Despite this, the common outcome observed across these studies is that CPS1 inhibition leads to a significant increase in intracellular ammonia levels. Therefore, inhibiting CPS1 expression in tumor cells may cause excessive ammonia accumulation, but whether this accumulation can induce ammonia-induced cell death in tumor cells requires further validation through comprehensive experimental studies.

## 5 Clinical relevance of ammonia death

### 5.1 Targeting glutamine metabolism-related pathways

Based on current research progress, anti-tumor strategies targeting glutamine metabolism have established a multi-level intervention system, primarily encompassing three major directions: transport inhibition, key enzyme blockade, and combination therapies. At the level of glutamine transport, SLC1A5, as a key transporter, has emerged as an important target. Preclinical studies have confirmed that IMD-0354 significantly suppresses the growth of melanoma and hepatocellular carcinoma (HCC) through competitive inhibition of SLC1A5 (152, 153). Similarly, V-9302, which also targets this transporter, has demonstrated anti-tumor activity in models of triple-negative breast cancer (TNBC), HCC, and melanoma. Its mechanism of action involves blocking glutamine uptake by tumor cells, thereby disrupting metabolic homeostasis (60, 154, 155). JHU083, a novel SLC1A5 inhibitor, has been shown to be effective against various solid tumors in animal experiments, particularly exhibiting potential in regulating the metabolic reprogramming of TAMs (76, 156).

Multiple inhibitors targeting glutaminase (GLS), a key enzyme in glutaminolysis, are currently in various stages of development. BPTES, a selective GLS inhibitor, has been shown to suppress the growth of multiple solid tumors in preclinical studies (157, 158). CB-839 (also known as Telaglenastat), one of the more advanced candidates, has entered clinical trials for the treatment of various solid tumors (156, 159). Other GLS inhibitors, such as IACS-6274 in head and neck squamous cell carcinoma (HNSCC) and IPN60090 in non-small cell lung cancer (NSCLC) and ovarian cancer, have demonstrated efficacy in preclinical models (160) (161). These agents inhibit the conversion of glutamine to glutamate, thereby blocking α-KG production, disrupting TCA cycle replenishment and biosynthesis, and ultimately leading to metabolic collapse in tumor cells.

Combination therapy strategies aim to overcome the limitations of monotherapy and enhance therapeutic efficacy. Preclinical studies have demonstrated that DRP-104, a glutamine mimetic prodrug, in combination with the MEK inhibitor trametinib, effectively suppresses pancreatic ductal adenocarcinoma by simultaneously inhibiting glutamine metabolism and the ERK signaling pathway (162, 163). Furthermore, progress has been made in targeting compensatory mechanisms employed by tumor cells under glutamine deprivation. For instance, IFRD1 knockout combined with CB-839 treatment effectively inhibited the “low-cost” survival of tumor cells under nutrient stress in HCC animal models by blocking the autophagic degradation pathway of histone H1.0 (164). Other strategies, such as targeting the RAS signaling pathway to suppress its mediated dysfunction in macrophage phagocytosis, remain at the theoretical exploration stage (165, 166). These multi-pathway, multi-target intervention strategies provide new research directions and therapeutic approaches for overcoming tumor metabolic heterogeneity and microenvironment-mediated drug resistance. (**Table 2**).

**Table 2:** Antitumor therapeutic strategies targeting glutamine metabolism-related pathways.

Therapeutic approach	Drugs	Mechanism of action	Research stage	Indications	References
Targeting Glutamine Transport	IMD-0354	Competitively inhibits SLC1A5	Preclinical research	Melanoma, HCC, etc.	(152, 153)
	V-9302	Competitively inhibits SLC1A5	Preclinical research	HCC, Melanoma, TNBC, etc.	(60, 154, 155)
	JHU083	Competitively inhibits SLC1A5	Animal experimental stage	Various solid tumors.	(76, 156)
Inhibiting GLS	BPTES	Selectively inhibits GLS	Preclinical research	Various solid tumors	(157, 158)
	CB-839	Selectively inhibits GLS	Clinical trial stage	Various solid tumors	(156, 159)
	IACS-6274	Selectively inhibits GLS	Preclinical research	HNSCC	(160)
	IPN60090	Selectively inhibits GLS	Preclinical research	NSCLC, ovarian cancer, etc.	(161)
Combination Therapies	DRP-104 + Trametinib	DRP-104 mimics glutamin; Trametinib inhibits ERK signaling pathway	Preclinical research	Pancreatic ductal adenocarcinoma	(162, 163)
	JHU083 + PD-1 inhibitors	Blocks glutamine metabolism, synergizes with PD-1 inhibitor	Animal experimental stage	Various solid tumors	(232)
	JHU083 + CAR-T	Enhances CAR-T cell persistence and anti-tumor activity via glutamine antagonism	Theoretical exploration stage	Various solid tumors	(233)
	IFRD1 knockout + CB-839	Inhibits IFRD1 protein; combines with GLS inhibitor	Animal experimental stage	HCC	(164)
Others	Targeting RAS signaling pathway	Inhibits RAS-mediated macrophage phagocytosis	Theoretical exploration stage	Various solid tumors	(165, 166)

BPTES, bis-2-(5-phenylacetamido-1,2,4-thiadiazol-2-yl) ethyl sulfide; DON, 6-diazo-5-oxo-L-norleucine; HCC, Hepatocellular carcinoma; NSCLC, non-small cell lung cancer; HNSCC, head and neck squamous cell carcinoma; TNBC, Triple Negative Breast Cancer.

### 5.2 Targeting ammonia death to promote tumor immunotherapy

#### 5.2.1 Ammonia death and adoptive T cell therapy

The concept of “ammonia death” in effector T cells, recently proposed by Professor Huang Bo’s team, offers valuable insights for improving T-cell-based cancer immunotherapy by enhancing the survival of Teff cells (15, 16). Their research demonstrates that targeting T-cell glutamine metabolism through drugs such as C381 or JHU083, or by adoptively transferring inducible GLS1 shRNA-expressing OT-I Teff cells into tumors, not only increases the number of OT-I Teff cells within the TME but also significantly reduces tumor growth and prolongs survival in animal models (15, 16). These findings suggest that blocking ammonia accumulation to improve effector T cell persistence may represent a promising new direction for advancing adoptive T-cell therapies aimed at tumors.

**Figure 5:**
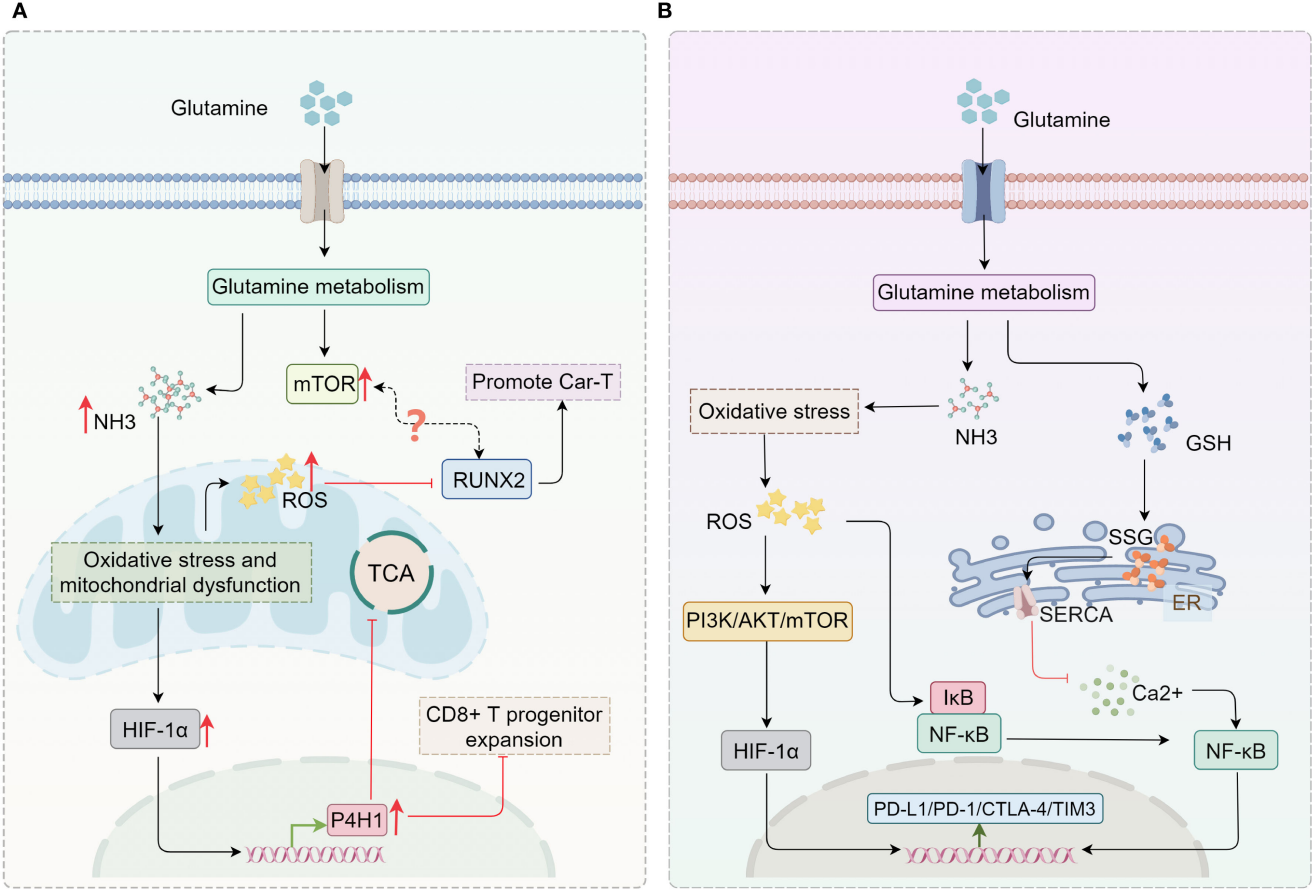
Potential association between ammonia accumulation and immunotherapy. **(A)** Ammonia accumulation resulting from glutamine metabolism can activate HIF-1α/P4HA1, thereby inhibiting the TCA cycle and the expansion of CD8+ T progenitor cells. Both ROS generated by oxidative stress and the mTOR pathway activated by glutamine metabolism can regulate RUNX2, further affecting CAR-T therapy. **(B)** Glutamine metabolism not only modulates the expression of immune checkpoints through the ROS-mediated PI3K/AKT pathway, but also influences the calcium/NF-κB signaling pathway by mediating GSH synthesis to regulate the expression of immune checkpoints. *GSH, glutathione; ER, endoplasmic reticulum; SERCA, sarcoendoplasmic reticulum calcium ATPase; SSG, protein S-glutathionylation; TCA, tricarboxylic acid; P4HA1, prolyl 4-hydroxylase 1.*.

CAR-T immunotherapy, one of the most innovative approaches in adoptive cell therapy, has shown considerable success in treating hematological malignancies (167, 168). However, the short lifespan of effector T cells remains a major limitation of CAR-T therapy (168, 169). This limitation directly impacts the long-term outcomes of treatment, particularly in terms of tumor relapse and sustained immune surveillance (168, 169). Studies have shown that during the early phase, ammonia derived from glutamine metabolism serves as a nitrogen source for CD8^+^ T cell proliferation and indirectly upregulates RUNX2 (a key transcription factor for osteoblast differentiation; RUNX2 overexpression enhances the antitumor efficacy of murine CAR-T cells and improves the function of human CAR-T cells) via the mTOR pathway (170–172). In the late phase, however, excessive ammonia accumulation induces lysosomal alkalization, collapse of mitochondrial membrane potential, and ROS burst, triggering oxidative stress and mitochondrial dysfunction. This subsequently activates the HIF-1α signaling pathway, leading to upregulation of P4HA1 (a critical regulator of CD8^+^ T cell differentiation that is highly upregulated in tumor-draining lymph nodes (TDLNs) and hypoxic tumor microenvironments; inhibition of P4HA1 enhances the expansion of progenitor CD8^+^ T cells while alleviating exhaustion, thereby boosting systemic antitumor immunity). Concurrently, ammonia inhibits the catalytic function of P4HA1 by chelating iron and competing with 2-oxoglutarate (2OG), thereby accelerating T cell exhaustion (173–175). Moreover, intracellular ammonia overload appears to downregulate RUNX2 by promoting ROS accumulation (176–178) (**Figure 5A**).

This time-dependent paradoxical effect of ammonia accumulation can be harnessed through precise interventional strategies to achieve therapeutic benefits. During the early stage of ammonia accumulation, maintaining an appropriate ammonia level supports RUNX2-mediated T cell function. For instance, transient early inhibition of ammonia production using glutaminase inhibitors (e.g., JHU083) can delay the exhaustion phenotype (15, 16). In the late phase, ammonia scavengers (e.g., C381, which lowers lysosomal pH) or overexpression of urea cycle enzymes (e.g., CPS1) can mitigate lysosomal damage and mitochondrial dysfunction, while inhibition of the HIF-1α/P4HA1 axis helps sustain T cell persistence (173, 179, 180). These findings suggest that suppressing ammonia accumulation in immune cells or targeting ammonia-induced cell death pathways may represent a promising strategy to prolong Teff survival and optimize CAR-T therapy. In this context, combining targeted ammonia metabolism intervention (e.g., using the GLS1 inhibitor CB-839) with CAR-T cell infusion not only blocks tumor cell utilization of ammonia but also enhances T cell adaptability through metabolic plasticity, thereby simultaneously alleviating the immunosuppressive microenvironment and prolonging CAR-T cell persistence (156, 159). This strategy integrates the dual roles of ammonia into a novel paradigm of “spatiotemporal regulation–metabolic intervention–immune synergy,” offering a new approach to overcome the limitations of CAR-T therapy.

#### 5.2.2 Targeting ammonia death to enhance ICI efficacy

ICIs have emerged as revolutionary therapeutic strategies in tumor immunotherapy, primarily by activating T cells and alleviating immunosuppressive signals (3, 181). However, T cells may still experience exhaustion during prolonged immune responses (182). Thus, prolonging the survival of effector T cells is a critical strategy to enhance the long-term efficacy of ICIs. Additionally, immune evasion remains a major challenge in ICI therapy. Despite the impressive success of immune checkpoint inhibitors across various tumor types, tumor cells can circumvent immune system attacks through several mechanisms, undermining the therapeutic potential of ICIs (183, 184). For example, tumor cells can evade immune surveillance not only through the PD-L1/PD-1 pathway but also by suppressing T cell function through other immune checkpoints such as TIM-3 and CTLA-4 (183, 184).

Research suggests that ammonia accumulation can trigger intracellular metabolic disorders and oxidative stress, such as increased ROS production, which activates key signaling pathways, including NF-κB, PI3K/AKT/mTOR, and HIF-1α (178, 185, 186). These pathways are integral to cellular responses to metabolic stress, oxidative stress, and immune reactions. Their activation is closely associated with immune evasion, tumor progression, and the regulation of immune cell function (186, 187).

The interaction between ROS and NF-κB is a critical mechanism of immunosuppression in chronic inflammation and the tumor microenvironment (188). Ammonia accumulation in CD8+ T cells triggers PD-1 upregulation via ROS/NF-κB (189, 190), while scavenging ammonia augments PD-1 blockade response *in vivo (* 191). Studies indicate that blocking glutamine metabolism in tumor cells can enhance the expression of PD-L1, enabling tumor cells to evade T cell-mediated immune killing (191, 192). Under glutamine-restricted conditions, intracellular GSH levels decrease, disrupting the activity of the sarco/endoplasmic reticulum calcium ATPase (SERCA) and activating the calcium/NF-κB signaling pathway. This activation leads to the translocation of NF-κB to the nucleus, where it binds directly to the PD-L1 promoter, promoting its transcription (191, 192). An analysis of clinical cohort data similarly demonstrated that the glutamine antagonist JHU083 promotes the proliferation of CD8^+^ T cells and enhances the efficacy of PD-1 inhibitors (193).

While blocking glutamine metabolism in combination with PD-1/PD-L1 inhibitors significantly boosts T cell anti-tumor activity *in vitro* and *in vivo* in tumor cells with primary resistance (low PD-L1 expression) (191), this approach may not be effective for immunotherapy in “hot” tumors that already exhibit high PD-L1 expression. Currently, detailed experimental studies on the regulatory role of ammonia accumulation in immune checkpoint expression in tumors are limited, and further research is needed. Nevertheless, evidence suggests that in TAMs and MDSCs, oxidative stress can activate IκB kinase (IKK) by promoting ROS production, leading to the degradation of IκB protein, the release of NF-κB, upregulation of PD-L1, and ultimately reduced anti-tumor T cell activity (194, 195). Similarly, under oxidative stress, ROS can inhibit PTEN, a negative regulator of the PI3K pathway, leading to the activation of the PI3K-AKT signaling axis (196, 197). AKT activation increases metabolic activity and transcriptional regulation *via* the mTOR pathway, which indirectly upregulates immune checkpoint molecules such as PD-1 and CTLA-4 (198, 199). HIF-1α, a key mediator of cellular responses to hypoxia and metabolic stress, is also activated by oxidative stress. By increasing ROS levels, oxidative stress can activate the PI3K/AKT and mTOR pathways, promoting the expression and stabilization of HIF-1α. The upregulation of HIF-1α induces T cells to express PD-1 and other exhaustion-related genes such as TIM-3 (200, 201) (**Figure 5B**). These findings suggest a potential link between ammonia accumulation and immune checkpoint expression, implying that ammonia accumulation can promote immune checkpoint expression in immune cells, leading to tumor drug resistance and immune evasion. Thus, targeting ammonia accumulation or ammonia death-related pathways in immune cells may provide a promising combination strategy to enhance the efficacy of ICIs.

## 6 Challenges and future directions of ammonia death in cancer therapy

Current strategies targeting ammonia-induced cell death face multiple challenges. Insufficient targeting specificity represents a core bottleneck: although glutaminase inhibitors (e.g., CB-839) can reduce ammonia accumulation in CD8^+^ T cells, their concurrent suppression of glutamine metabolism in tumor cells may exacerbate nutrient competition within the immune microenvironment, thereby impairing T-cell function (127, 202). Another major obstacle is the ammonia tolerance exhibited by tumor cells. Studies have shown that HCC and pancreatic cancer, among others, highly express CPS1, a urea cycle enzyme that promotes survival by enhancing ammonia detoxification (135, 203). Furthermore, the heterogeneity of the immune microenvironment further limits therapeutic efficacy: competitive uptake of glutamine by tumor cells via SLC38A2 significantly impairs the antigen-presenting capacity of dendritic cells (33), while MDSCs and TAMs exhibit enhanced immunosuppressive activity under glutamine-deficient conditions (79). Inadequate drug delivery efficiency is also a concern; small-molecule inhibitors such as V-9302, due to their lack of tumor targeting, may disrupt metabolic homeostasis in normal tissues (204).

Potential side effects also warrant serious consideration. Preclinical studies indicate that systemic glutamine deprivation may induce muscle atrophy and intestinal mucosal damage (7), while high-dose administration of the ammonia scavenger sodium phenylbutyrate carries a risk of neurotoxicity (128). Particularly noteworthy is that inhibiting ammonia clearance may inadvertently promote tumor progression: ammonia drives lipid synthesis by activating SREBP-1, and impaired ammonia clearance may accelerate tumor metastasis (90).

Future research should focus on four major breakthrough directions: (1). Spatiotemporally precise regulation: Develop tumor microenvironment-responsive prodrugs (e.g., DRP-104) to achieve localized induction of ammoniagenesis, thereby avoiding systemic toxicity. (2). Combined metabolic intervention: The combination of CPS1 inhibitors and GLS inhibitors may simultaneously disrupt tumor ammonia detoxification barriers and alleviate ammonia toxicity in CD8^+^ T cells. (3). Immunometabolic reprogramming: Enhancing SLC38A2 expression in type 1 conventional dendritic cells (cDC1s) may improve glutamine uptake and potentially reverse antigen presentation defects. (4). Dynamic monitoring techniques: Utilize positron emission tomography (PET) imaging with ¹¹C-labeled BPTES to monitor tumor glutaminase activity in real time for guiding precision drug administration. Special emphasis should be placed on elucidating the dual regulatory mechanisms of ammonia metabolism in CAR-T therapy: maintaining appropriate ammonia levels in the early phase to activate RUNX2-mediated T-cell function, while reducing lysosomal pH via agents such as C381 in the later phase to delay T-cell exhaustion (172).

## 7 Conclusion

Ammonia death, a newly identified form of T cell death, has been shown to play a key role in the demise of CD8+ T cells (15, 16). In the TME, activated CD8+ T cells undergo rapid proliferation and metabolic reprogramming to sustain their anti-tumor activity (59, 60). This reprogramming accelerates glutamine metabolism, producing large amounts of ammonia as a byproduct, which accumulates intracellularly and triggers cell death (15, 16). However, the TME is a highly complex regulatory network, encompassing not only CD8+ T cells but also other immune cells and tumor cells (57). This review examines glutamine metabolism in various immune and tumor cells within the TME and finds that elevated glutamine metabolic activity is not exclusive to CD8+ T cells. The activation of other immune cells also involves glutamine metabolism, and in particular, evidence suggests that glutamine metabolism in tumor cells may be even more pronounced than in immune cells. Therefore, regulating ammonia death-related pathways could potentially benefit other immune cells, while ammonia accumulation may also influence tumor cells themselves. These interactions warrant further investigation and exploration.

Inhibiting pathways associated with ammonia-induced cell death can enhance CD8+ T-cell survival, significantly improving the effectiveness of adoptive cell therapy (15). However, the precise role of ammonia in this process remains unclear. While previous research suggests that ammonia accumulation may indirectly influence potential CAR-T therapy targets and modulate the expression of immune checkpoints through the activation of oxidative stress, experimental validation is still needed. Elucidating the mechanisms of ammonia conversion and clearance within the TME could pave the way for new therapeutic strategies aimed at mitigating ammonia-induced damage in immune cells while promoting ammonia-induced cell death in tumor cells.

Current targeting strategies continue to confront a triple paradox: First, while inhibition of glutamine metabolism enhances memory differentiation of T cells (60), it may exacerbate nutrient competition within the tumor microenvironment. Second, although ammonia scavengers prolong T cell survival (104), they may promote tumor lipid synthesis via activation of the SREBP-1 pathway (90). Third, the “double-edged sword effect” of ammonia in CAR-T therapy—supporting T cell priming at early stages while accelerating exhaustion in later phases (172). Overcoming these challenges requires spatiotemporally dynamic regulatory strategies: maintaining appropriate ammonia levels during early treatment to promote T cell function, while controlling toxicity in later stages through lysosomal pH modulators (e.g., C381) or urea cycle enzyme activators.

Future research should focus on four dimensions: (1). In-depth mechanistic investigation: Clarifying the molecular switches of ammonia-induced death in other immune cells such as CD4^+^ T cells and TAMs. (2). Remodeling the metabolic microenvironment: Developing bifunctional nanocarriers targeting tumor ammonia metabolism (e.g., PD-L1 antibody conjugates loaded with JHU083). (3). Clinical translation exploration: Validating the efficacy of combinations such as DRP-104 with immune checkpoint blockers in specific subgroups, for instance, KEAP1-mutant lung cancer (163). (4). Technological innovation: Utilizing single-cell metabolomics to delineate spatiotemporal landscapes of ammonia transport (e.g., regulatory mechanisms of RHCG channels). Only through multi-omics integration and precise intervention can ammonia-induced cell death be transformed from a “stumbling block” into a “stepping stone” for immunotherapy, ultimately breaking through the current survival bottlenecks in tumor immunotherapy.

In conclusion, inhibiting ammonia death in effector CD8+ T-cells theoretically holds promise for improving immunotherapy efficacy. However, the underlying mechanisms of ammonia death in CD8+ T-cells remain under investigation, and mechanisms in other cell types are yet to be explored. Continued research into the immunological aspects of ammonia metabolism in the TME is essential for advancing tumor immunotherapy targeting ammonia death, with the goal of achieving innovative breakthroughs in cancer treatment and providing clinical benefits to cancer individuals.
